# Inadvertent Worsening of a Small Primary Spontaneous Pneumothorax: A Case Report Highlighting the Importance of Adhering to British Thoracic Society (BTS) Guidelines

**DOI:** 10.7759/cureus.80043

**Published:** 2025-03-04

**Authors:** Michael Shakhloul, Ahmed Amer

**Affiliations:** 1 Emergency Medicine, Royal Surrey County Hospital, Guildford, GBR

**Keywords:** chest aspiration, chest drain, emergency procedures, seldinger chest tube, spontaneous pneumothorax

## Abstract

We present a case of a 21-year-old male patient who attended the Emergency Department (ED) with chest pain. Initial investigations appeared normal, leading to discharge. Subsequently, radiological reporting identified a small left-sided pneumothorax, prompting recall. Despite being clinically stable, the patient underwent unnecessary aspiration, which exacerbated the pneumothorax, resulting in partial lung collapse. A chest drain was subsequently required. This case highlights the importance of adherence to British Thoracic Society (BTS) guidelines in managing spontaneous pneumothorax to avoid unnecessary interventions and complications.

## Introduction

Primary spontaneous pneumothorax (PSP) occurs due to the presence of air in the pleural space without an apparent external cause, typically in young, thin males and smokers, even in the absence of underlying lung disease. The incidence of PSP is estimated at 18-28 cases per 100,000 per year in men and 1.2-6 cases per 100,000 per year in women [1]. Management of PSP is guided by the British Thoracic Society (BTS) 2023 guidelines, which advocate for conservative management in stable patients with small pneumothoraces (<2 cm at the hilum or <3 cm at the apex) [2]. The 2020 New England Journal of Medicine (NEJM) PSP trial also demonstrated that conservative treatment could avoid unnecessary interventions while maintaining similar long-term outcomes [3]. This case highlights the clinical consequences of deviating from these guidelines, emphasizing the risks of unwarranted procedures leading to complications. It underscores the importance of strict adherence to evidence-based guidelines and effective clinical handover practices to optimize patient outcomes.

## Case presentation

A 21-year-old male presented to the Emergency Department (ED) with mild, left-sided chest pain. He had no medical history, trauma, or significant risk factors. The initial clinical examination was unremarkable. ECG, blood tests, and chest X-ray (CXR) were initially interpreted as normal. He was discharged with musculoskeletal chest pain as the presumed diagnosis.

**Figure 1:**
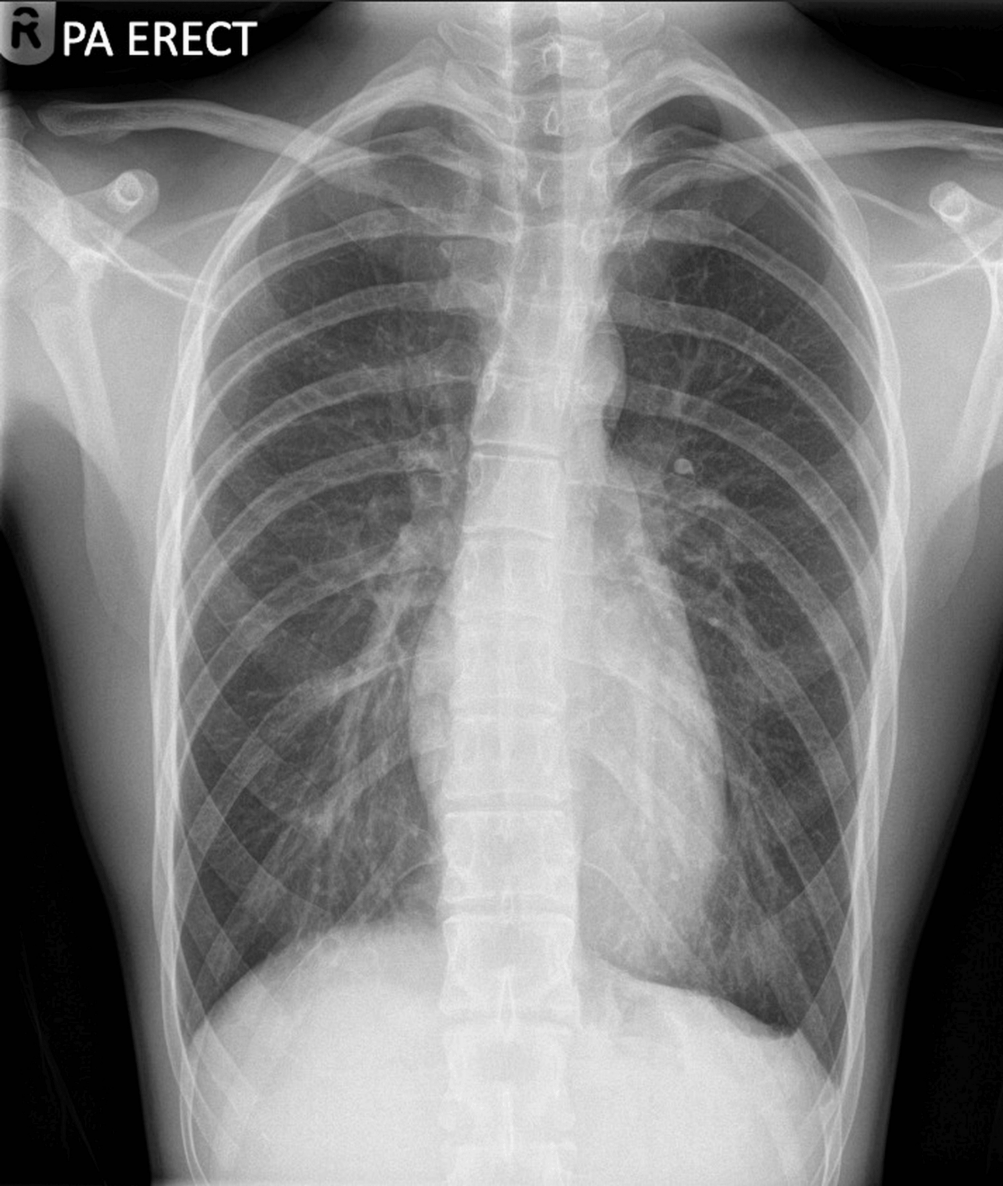
Initial CXR demonstrating a small apical left-sided pneumothorax. CXR, chest X-ray

The next day, formal radiology reporting revealed a small apical left-sided pneumothorax (Figure 1), and the patient was called back for reassessment. At re-evaluation, he was completely asymptomatic with stable vital signs. A repeat CXR showed no change in pneumothorax size. Despite guidelines recommending conservative management, the consultant decided on needle aspiration.

**Figure 2:**
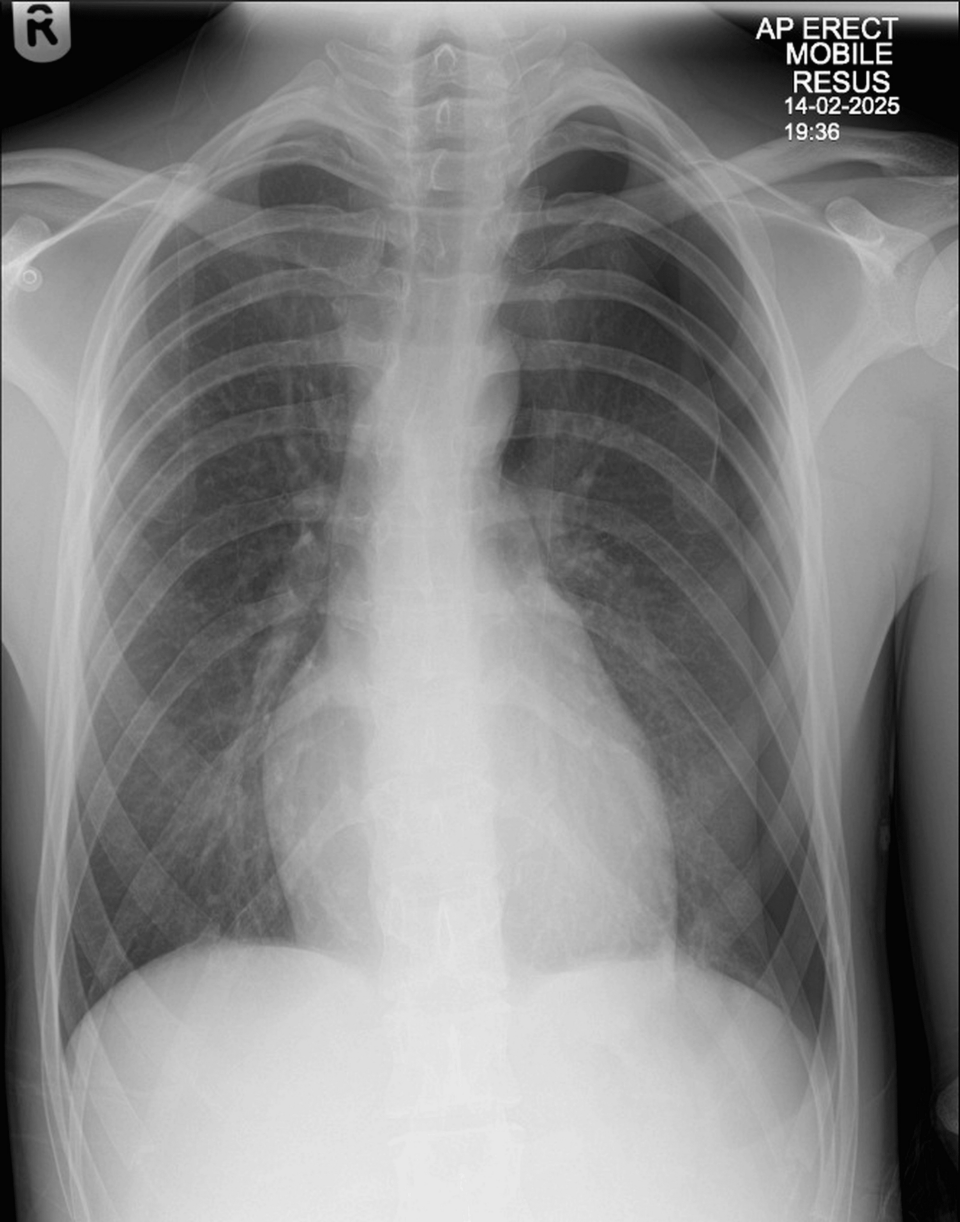
CXR following needle aspiration demonstrating enlargement of pneumothorax and partial lung collapse. CXR, chest X-ray

Following aspiration, the patient's repeat CXR unexpectedly showed increased pneumothorax size with partial lung collapse (Figure 2).

**Figure 3:**
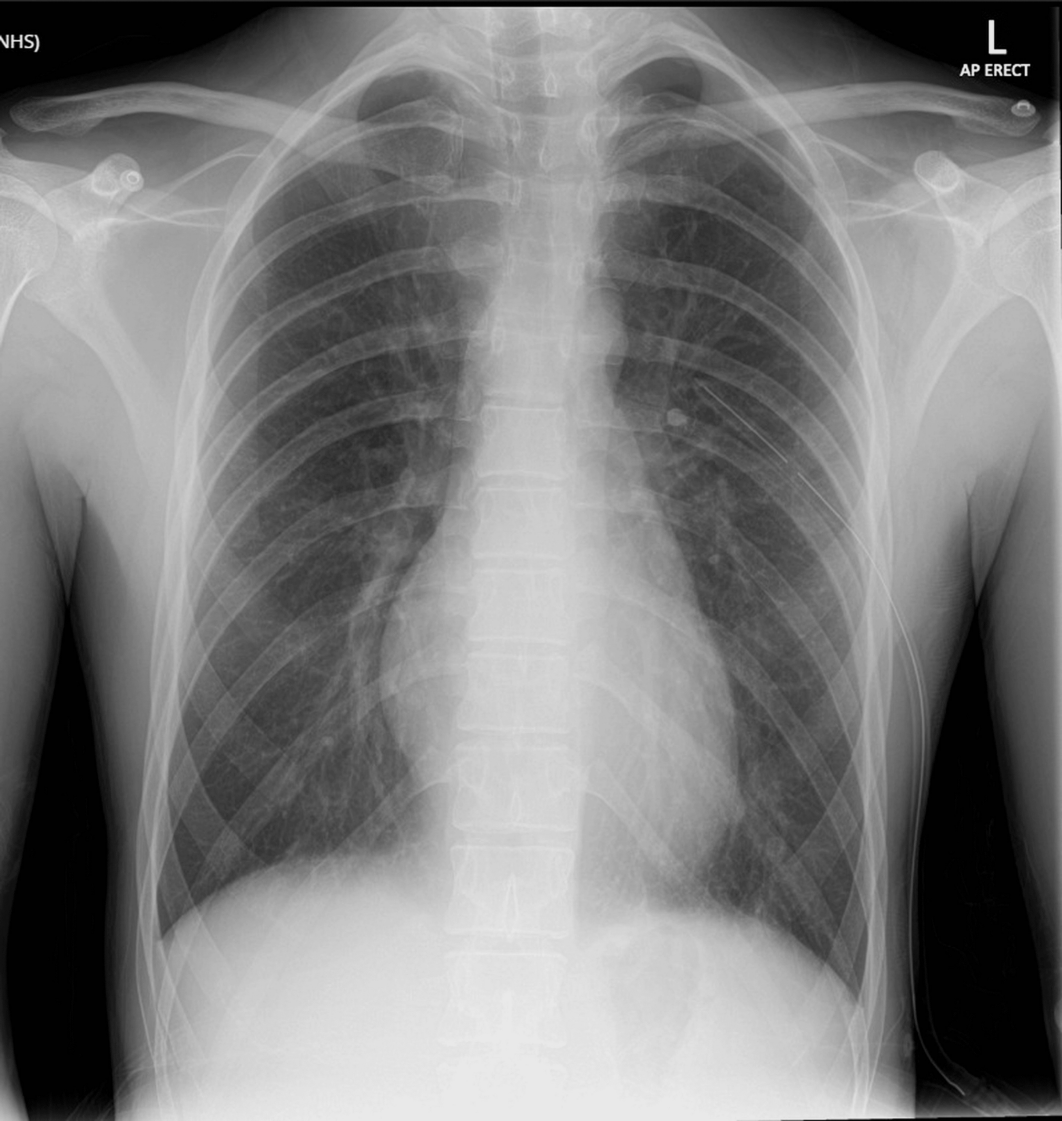
CXR after chest tube insertion demonstrating lung re-expansion and correct tube position. CXR, chest X-ray

At shift handover, the incoming consultant initially declined chest drain insertion. After further discussion, the potential risk of tension pneumothorax overnight was acknowledged, and a chest tube was inserted. The repeat CXR showed correct chest tube placement and successful lung re-expansion (Figure 3). The patient remained stable overnight and was referred to respiratory medicine for ongoing inpatient management and follow-up.

## Discussion

According to the BTS guidelines, small (<2 cm), asymptomatic PSP should be managed conservatively with observation. Needle aspiration is reserved for larger or symptomatic pneumothoraces [1-3]. In this case, deviation from these guidelines led directly to complications. Aspiration caused an increase in pneumothorax size and partial lung collapse, necessitating the insertion of a chest drain, which is a more invasive procedure and carries additional risks, including infection, pain, and a prolonged hospital stay [4].

This case also illustrates the challenge of clinical handovers, where conflicting management decisions may arise. Clear communication and early escalation are critical to patient safety. Encouraging adherence to guidelines minimizes the risk of procedural complications and improves patient outcomes.

## Conclusions

Adherence to established guidelines such as those from the BTS is critical to prevent complications and unnecessary interventions in spontaneous pneumothorax management. This case underscores the importance of evidence-based decision-making and clear communication between clinical teams.
